# COX-2 inhibition is neither necessary nor sufficient for celecoxib to suppress tumor cell proliferation and focus formation in vitro

**DOI:** 10.1186/1476-4598-7-38

**Published:** 2008-05-16

**Authors:** Huan-Ching Chuang, Adel Kardosh, Kevin J Gaffney, Nicos A Petasis, Axel H Schönthal

**Affiliations:** 1 Department of Biochemistry & Molecular Biology, University of Southern California, Los Angeles, USA; 2 Department of Molecular Microbiology & Immunology, University of Southern California, Los Angeles, USA; 3 Department of Chemistry, University of Southern California, Los Angeles, USA

## Abstract

**Background:**

An increasing number of reports is challenging the notion that the antitumor potential of the selective COX-2 inhibitor celecoxib (Celebrex^®^) is mediated primarily via the inhibition of COX-2. We have investigated this issue by applying two different analogs of celecoxib that differentially display COX-2-inhibitory activity: the first analog, called unmethylated celecoxib (UMC), inhibits COX-2 slightly more potently than its parental compound, whereas the second analog, 2,5-dimethyl-celecoxib (DMC), has lost the ability to inhibit COX-2.

**Results:**

With the use of glioblastoma and pancreatic carcinoma cell lines, we comparatively analyzed the effects of celecoxib, UMC, and DMC in various short-term (≤48 hours) cellular and molecular studies, as well as in long-term (≤3 months) focus formation assays. We found that DMC exhibited the most potent antitumor activity; celecoxib was somewhat less effective, and UMC clearly displayed the overall weakest antitumor potential in all aspects. The differential growth-inhibitory and apoptosis-stimulatory potency of these compounds in short-term assays did not at all correlate with their capacity to inhibit COX-2, but was closely aligned with their ability to trigger endoplasmic reticulum stress (ERS), as indicated by the induction of the ERS marker CHOP/GADD153 and activation of the ERS-associated caspase 7. In addition, we found that these compounds were able to restore contact inhibition and block focus formation during long-term, chronic drug exposure of tumor cells, and this was achieved at sub-toxic concentrations in the absence of ERS or inhibition of COX-2.

**Conclusion:**

The antitumor activity of celecoxib in vitro did not involve the inhibition of COX-2. Rather, the drug's ability to trigger ERS, a known effector of cell death, might provide an alternative explanation for its acute cytotoxicity. In addition, the newly discovered ability of this drug to restore contact inhibition and block focus formation during chronic drug exposure, which involved neither ERS nor COX-2, suggests a novel, as yet unrecognized mechanism of celecoxib action.

## Introduction

Celecoxib (CXB) has been developed as a selective inhibitor of cyclooxygenase-2 (COX-2) and is widely prescribed under the trade name Celebrex^® ^for relief of symptoms of osteoarthritis and rheumatoid arthritis; it was also approved as an adjunct to the standard of care for patients with familial adenomatous polyposis (FAP) [1]. In the laboratory, CXB has demonstrated anti-cancer activity in various animal tumor models, and it is suspected that this drug might be useful for the prevention and treatment of colorectal and possibly other types of cancer as well [2-4]. Indeed, several clinical trials have demonstrated CXB's potency to reduce colorectal polyp and adenoma formation in humans [1,5,6]. However, these promising results were overshadowed by concomitantly emerging side effects, such as life-threatening cardiovascular complications, which are thought to be caused by the long-term inhibition of COX-2 (reviewed in [7]).

The underlying molecular mechanisms by which CXB exerts its anti-tumor effects have become controversial, primarily due to an increasing number of reports describing effects of this drug that appear to take place in the absence of any apparent involvement of COX-2 (see refs. [8-10] for review). In this regard, several non-COX-2 components of the cell have been identified and proposed as candidates for mediating the COX-2-independent antitumor effects of CXB. One of the best studied and perhaps most relevant of these alternative targets is sarcoplasmic/endoplasmic reticulum (ER) calcium ATPase (SERCA) [11-13], which is an ER transmembrane protein that is responsible for pumping calcium from the cytosol into the ER, thereby maintaining the steep gradient of this ion between the two subcellular compartments.

Inhibition of SERCA represents the earliest detectable effect of drug action, as increased cytosolic calcium levels can be measured within seconds of adding CXB to cultured cells [11-15]. Leakage of calcium from the ER has long been known to act as a potent trigger of ER stress, and ER stress-instigated cell death can indeed be verified after CXB treatment of tumor cells in vitro and in animal tumor models in vivo [12,16-19]. Known critical executioners of the pro-apoptotic arm of the ER stress response are, for example, CHOP/GADD153 (CCAAT/enhancer binding protein homologous transcription factor/growth arrest and DNA damage-inducible gene 153) and caspase 7, both of which are strongly stimulated by CXB treatment (for example, refs. [16,17,19-21]. Altogether, the discovery of SERCA and subsequent ER stress as a direct target of CXB, in combination with a host of other observations [8], has seriously challenged the notion that inhibition of COX-2 might be the critical event mediating the antitumor outcomes of CXB treatment [10,22].

In an effort to shed further light on this controversy, we have investigated the in vitro antitumor potential of two close structural analogs of CXB with either increased or greatly decreased COX-2-inhibitory activity. The first analog, unmethylated-celecoxib (UMC), harbors 20% greater COX-2-inhibitory potency than CXB. The second analog, 2,5-dimethyl-celecoxib (DMC), lacks COX-2-inhibitory potency. In this report, we show that the antitumor potency of these compounds is DMC>CXB>UMC, i.e., the antitumor effects of these drugs are in inverse relation to their COX-2-inhibitory potential, but are closely aligned with their ability to trigger ER stress.

## Results

### CXB and UMC, but not DMC, inhibit COX-2 and block PGE_2 _biosynthesis

**Figure 1:**
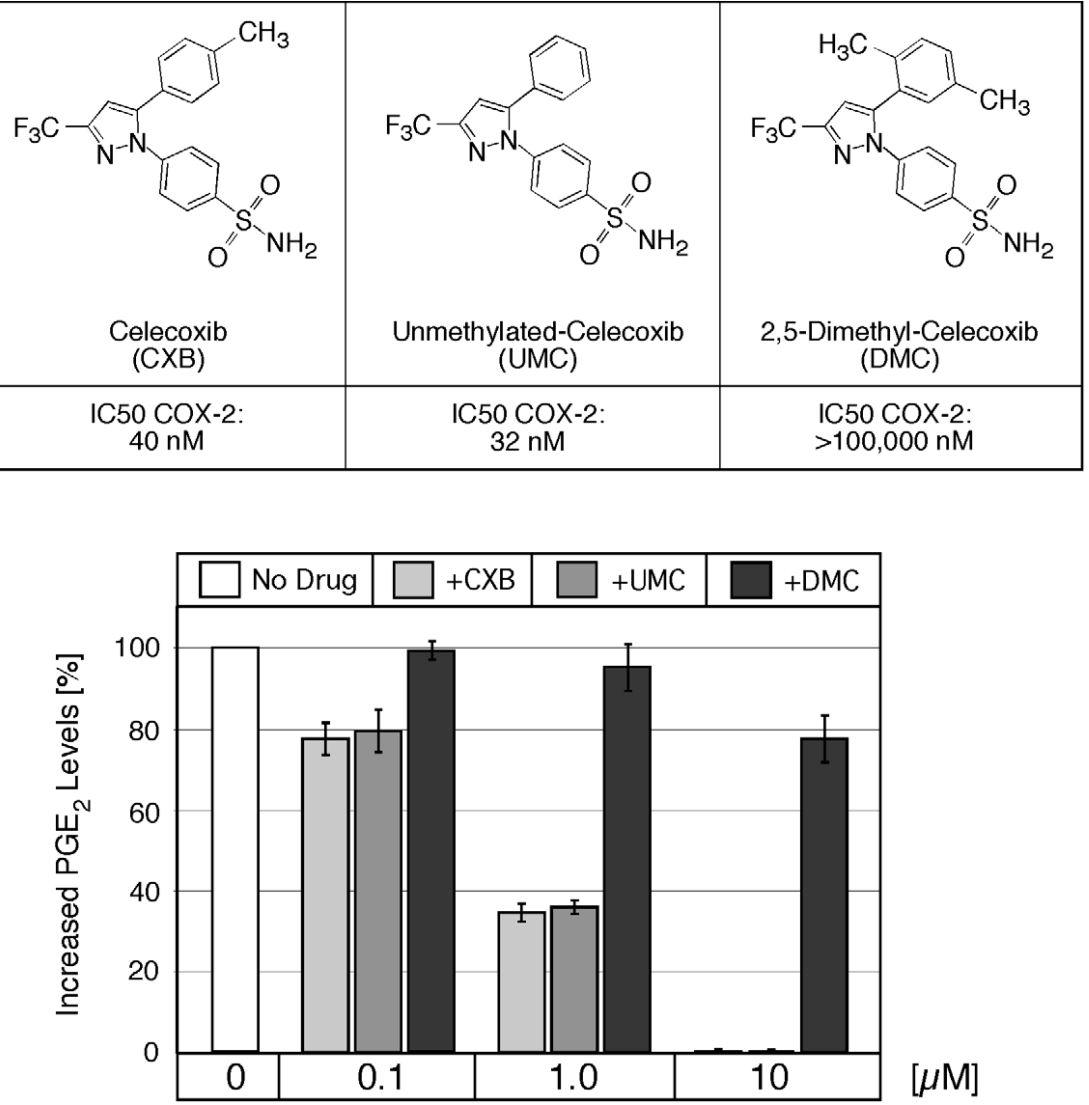
**Chemical structures and activities of CXB, UMC, and DMC**. CXB has one methyl group at the C-4 (p) position of its terminal phenyl ring; this substitution is lacking in UMC; DMC has two methyl groups (at the 2- and 5-positions). The listed COX-2 inhibitory potency (IC50) of these compounds is derived from two earlier studies that used human recombinant COX-2 in vitro [23, 42]. The bottom chart displays PGE_2 _levels (mean ± SE, n = 4) in the culture medium of Bx-PC-3 cells pretreated for 30 minutes with the indicated drug concentrations and then stimulated with 10 μM arachidonic acid, as previously described by Eibl et al. [25].

Figure 1 summarizes the chemical structures and properties of celecoxib (CXB) and its two analogs that were used in our study. CXB has one methyl group at the C-4 (p) position of its terminal phenyl ring; the methyl substitution is lacking in unmethylated-celecoxib (UMC); in contrast, 2,5-dimethyl-celecoxib (DMC) has two methyl groups (at the 2- and 5-positions) of the phenyl ring. The COX-2 inhibitory potency of these compounds was first described by Penning et al. [23], and is shown in Figure 1. Note that UMC is the most potent COX-2 inhibitor, followed by CXB, and DMC essentially is lacking any substantial COX-2-inhibitory potency.

Because the COX-2-inhibitory activity of the three compounds had been determined by in vitro assays with purified recombinant COX-2 enzyme [23], we next verified that these compounds exerted their activities also when added to cells in culture. For this purpose, we used a human pancreatic carcinoma cell line with high COX-2 expression levels and determined whether increasing concentrations of CXB, UMC, or DMC were able to block the synthesis of the COX-2 substrate prostaglandin E2 (PGE_2_). As shown in Figure 1, both CXB and UMC effectively prevented the formation of PGE_2 _with an IC50 below 1 μM and 100% inhibition at 10 μM. In contrast, DMC did not display substantial inhibitory activity; even at 10 μM, there was only a minor reduction (22%) of PGE_2 _biosynthesis. Together, these measurements confirmed that CXB and UMC were able to potently inhibit COX-2 in cell culture, and that DMC was much (at least 100-fold) less active.

### CXB, UMC, and DMC trigger ER stress and reduce cell viability at different potencies

**Figure 2:**
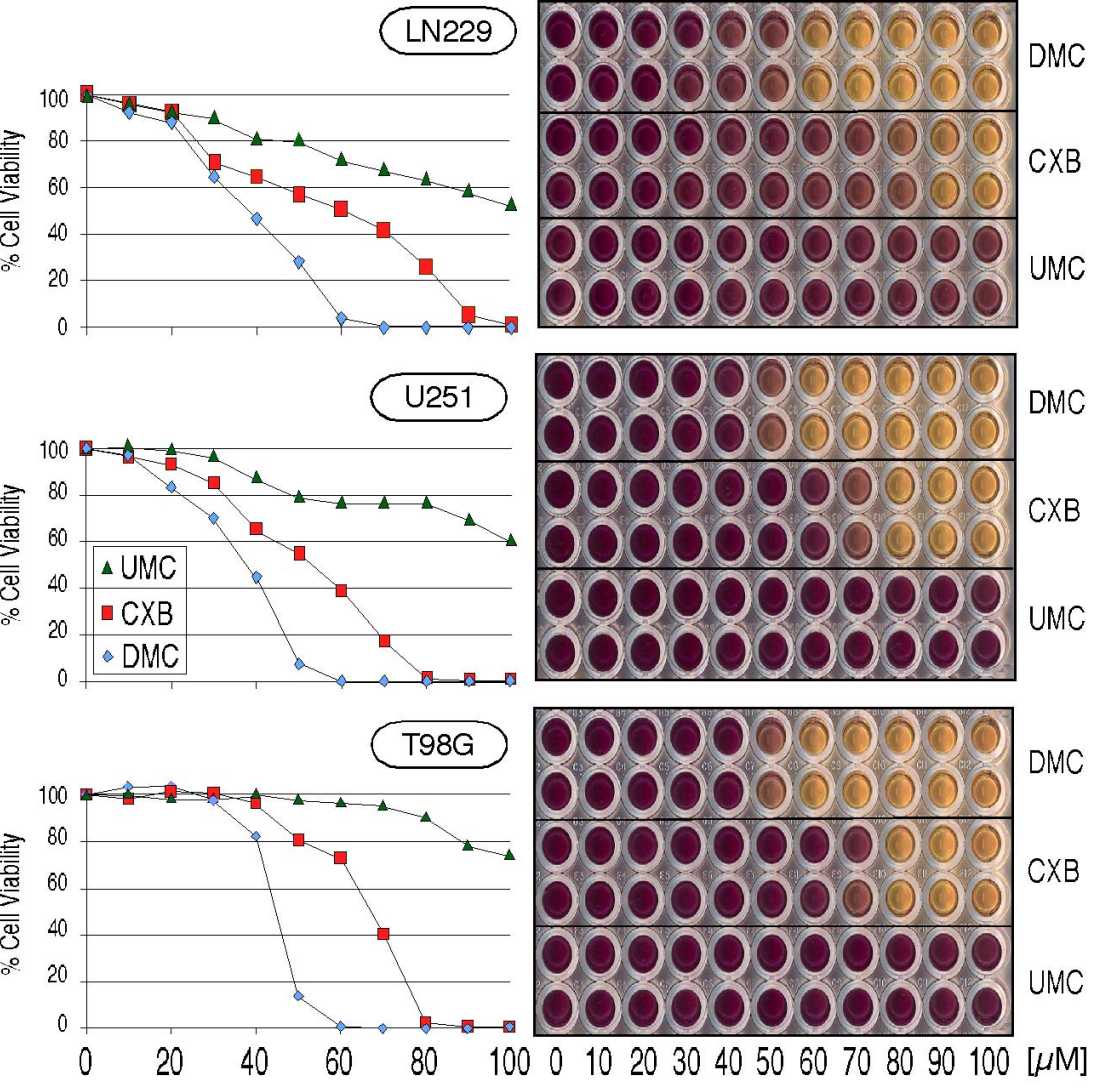
**Reduced cell growth and survival in the presence of CXB, UMC, and DMC**. Three different glioblastoma cell lines (LN229, U251, T98G) were treated with the indicated concentrations of CXB, UMC, or DMC. After 48 hours, MTT assays were performed to indicate the overall viability in response drug treatment. The right panels show representative 96-well plates of treated cells. Deep purple indicates fully viable cell cultures, whereas yellow color reveals drug cytotoxicity (each condition was tested in duplicate). The charts on the left present the quantitative readout of the optical density of the 96-well plates (average of two wells each). The whole experiment was independently repeated and yielded very similar outcomes.

To determine how CXB, UMC, and DMC would affect cell proliferation and survival, we treated three different glioblastoma cell lines (LN229, U251, and T98G) with increasing concentrations of these compounds and determined cell viability by MTT assay. As shown in Figure 2, DMC potently reduced cell viability with an IC50 in the range of 35–45 μM; CXB was slightly less potent with an IC50 of 55–65 μM. In comparison, UMC showed the weakest cytotoxic effects and its IC50 was >100 μM. When additional human tumor cell lines were tested, such as multiple myeloma, breast carcinoma, or lymphoma (all of which displayed greatly varying levels of COX-2 expression), very similar outcomes were observed (not shown). In all cases, DMC was the most cytotoxic compound, CXB was 20–30% weaker, and UMC was the weakest, exposing an obvious lack of correlation between the COX-2-inhibitory efficacy of these compounds and their cytotoxic potential. In addition, the exogenous addition of PGE_2 _was unable to overcome the growth-inhibitory effect of these drugs (not shown; see [24] for celecoxib), further indicating that alterations in PGE_2 _levels had no major consequence for cell proliferation in these cells.

**Figure 3:**
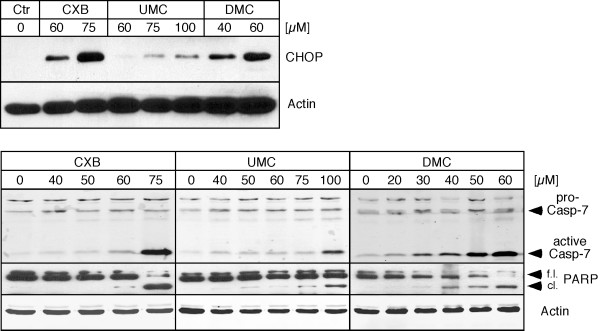
**Increased expression of markers for ER stress and apoptosis in response to treatment with CXB, UMC, and DMC**. U251 glioblastoma cells were treated with the indicated concentrations of CXB, UMC, and DMC and cell lysates were analyzed by Western blot with specific antibodies to CHOP (a pro-apoptotic ER stress indicator protein), cleaved (i.e., activated) caspase 7 (an ER stress-associated protein that participates in the execution of apoptosis), and PARP (proteolytic cleavage of PARP is executed by caspase 3 and indicates ongoing apoptosis). To verify equal loading in each case, the blots were also probed with an antibody to actin. The top panels represent lysates from cells treated with drugs for 18 hours (to reveal earlier events during ER stress). The bottom panels represent lysates from cells treated with drugs for 48 hours (to reveal later stages of ER stress-induced apoptosis). f.l.: full length PARP; cl.: cleaved PARP.

Because it was reported before that ER stress might be a critical mechanism by which celecoxib exerts its antitumor function, we investigated two established markers of ER stress, i.e., the pro-apoptotic protein CHOP and caspase 7. U251 glioblastoma cells were treated with different concentrations of CXB, UMC, and DMC, and cell lysates were analyzed by Western blot. As shown in Figure 3, all three drugs were able to stimulate CHOP expression levels and trigger activation of caspase 7. However, the respective effective concentrations differed substantially. In the case of CHOP induction, 40 μM DMC was as effective as 60 μM CXB, but UMC even at 100 μM displayed overall weaker effects. In the case of caspase 7 activation, 60 μM DMC was approximately as potent as 75 μM CXB, but UMC, again, even at 100 μM displayed weaker effects. In addition, we examined the cleavage of PARP (poly-ADP-ribose polymerase), which serves as an indicator of ongoing apoptosis. As before, DMC displayed the strongest effects on PARP cleavage; CXB was somewhat less potent and UMC exhibited only weak effects. Thus, overall, the effects of the three drugs on molecular markers of ER stress and apoptosis correlated with their cytotoxic potency (as shown in Figure 2), but not with their ability to inhibit COX-2.

### CXB, UMC, and DMC block focus formation at different potencies

Because the above assays were focused on rather short-term (up to 48 hours) drug effects, we decided to include additional experiments that allowed the evaluation of phenotypic responses during much longer periods of drug exposure (up to 3 months). In this regard, we chose focus formation assays, as these would enable the characterization of drug effects on a select tumor-specific feature and would exclude short-term cytotoxic effects that might mask other, more long-term consequences of drug treatment. The focus formation assays were performed at lower drug concentrations that were not readily toxic, but allowed the cells to form a confluent monolayer, which is a prerequisite for focus formation.

**Figure 4:**
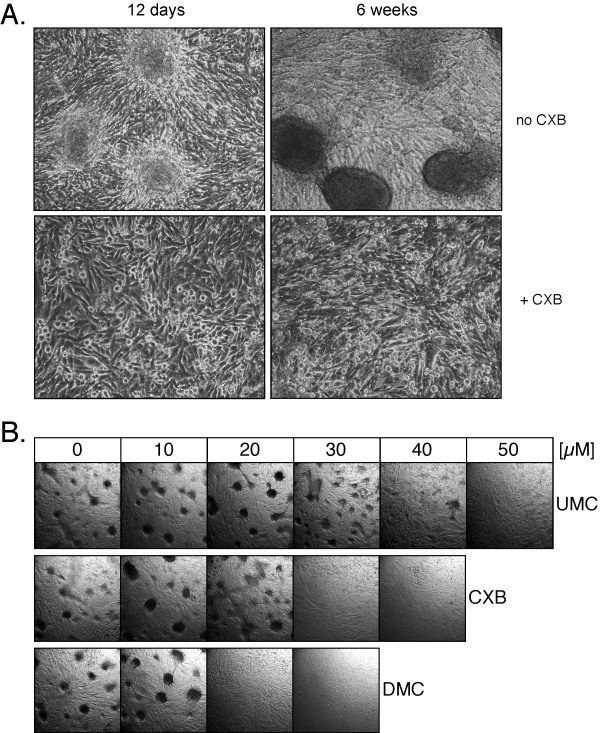
**Prevention of focus formation by CXB, DMC, and UMC**. U251 glioblastoma cells were continuously exposed to various concentrations of CXB, DMC, or UMC for up to 3 months in the same cell culture dishes (i.e., without splitting the cell monolayers). In A., cells were treated with or without 30 μM CXB and photomicrographs (160× magnification) were taken after 12 days and 6 weeks. Top left shows early-stage focus formation (3 representative foci are shown); top right shows 3 examples of fully developed, very compact foci (each one consisting of an estimated several hundred cells). Neither early-nor late-stage foci were present in CXB-treated cell cultures. In B., cells were treated with increasing concentrations of the three drugs and photomicrographs (20× magnification) were taken after 10 weeks. Note that in the absence of drug treatment, many foci (several hundred per square inch) had developed. However, no foci developed in the presence of 20 or 30 μM DMC, 30 or 40 μM CXB, and 50 μM UMC (representative sections of each culture are shown).

U251 glioblastoma cells were seeded at low density and were cultured in the continuous presence of increasing concentrations of CXB, UMC, or DMC for up to 3 months. Because the cell cultures were not split during this period, they formed dense monolayer cultures over time. In the absence of drug treatment, the cells began to aggregate and form foci after approximately 12 days; over the course of the following weeks, these foci developed into large, very tight spheres of compacted cells that were raised above the residual monolayer (Figure 4A). In contrast, in the presence of 30 μM CXB, focus formation was completely prevented (Figure 4A). Similarly, DMC and UMC were able to prevent focus formation, but differential drug concentrations were required. As shown in Figure 4B, the lowest effective concentrations were 20 μM for DMC, 30 μM for CXB, and 50 μM for UMC. It is noteworthy that these drug concentrations did not block tumor cell proliferation and did not prevent the formation of a complete monolayer, although the overall rate of cell proliferation was very slightly reduced, i.e., it took slightly longer for drug-treated cells to cover the entire surface of the culture dish. Nonetheless, even during extended culture in the presence of drugs (up to 3 months), no foci developed at the above mentioned drug concentrations, whereas in the absence of drug treatment, there were hundreds of foci per square inch. Thus, in summary, these results demonstrate that CXB, UMC, and DMC are able to suppress an important indicator of the transformed phenotype, focus formation, but this effect did not correlate with these drugs' potency to inhibit COX-2.

### CXB, UMC, and DMC display their differential antitumor activities in cells with or without COX-2 expression

**Figure 5:**
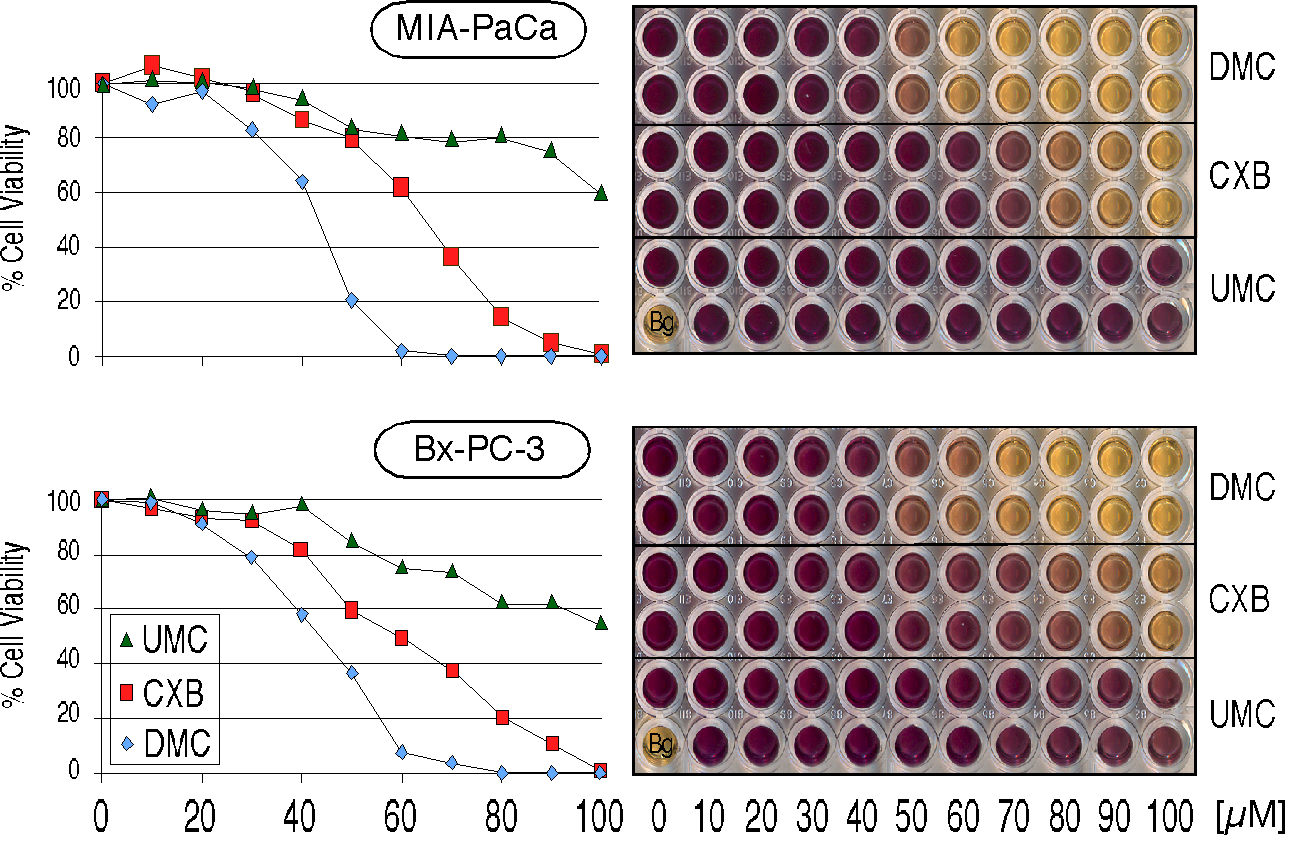
**Reduced cell growth and survival by CXB, UMC, and DMC in tumor cells with high or no COX-2 expression**. MIA-PaCa-2 (COX-2 negative) and Bx-PC-3 (high COX-2 expression) pancreatic carcinoma cells were treated with the indicated concentrations of CXB, UMC, or DMC. After 48 hours, MTT assays were performed to indicate the overall viability in response drug treatment. The right panels show representative 96-well plates of treated cells. Deep purple indicates fully viable cell cultures, whereas yellow color reveals drug cytotoxicity (each condition was tested in duplicate). The single well labeled "Bg." represents background of medium alone without cells. The charts on the left present the quantitative readout of the optical density of the 96-well plates (average of two wells each). The whole experiment was independently repeated and yielded very similar outcomes.

To further investigate the above mechanisms, we applied CXB, UMC, and DMC side-by-side to a pair of pancreatic carcinoma cell lines that had been well characterized previously with regards to their COX-2 status: MIA-PaCa-2 cells are negative for COX-2 expression, whereas Bx-PC-3 cells express high levels of COX-2 [25,26]. Both cell lines were exposed to increasing concentrations of the drugs, and MTT assays were performed 48 hours later. As shown in Figure 5, both cell lines responded similarly to CXB, UMC, and DMC. As before in the case of glioblastoma cell lines (Figure 2), DMC turned out to be the most potent cytotoxic compound (IC50 ~45 μM), CXB was noticeably weaker (IC50 60–65 μM), and UMC was the least effective (IC50>100 μM). Thus, the status of COX-2 expression did not have an apparent influence on cellular sensitivity to these drugs.

**Figure 6:**
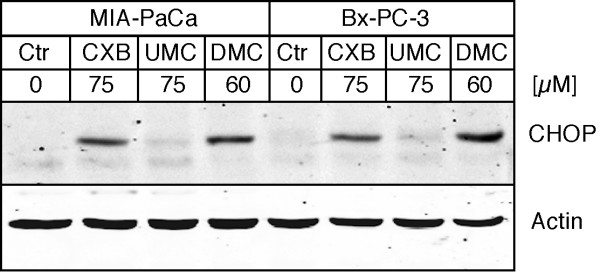
**Increased expression of ER stress marker CHOP by CXB, UMC, and DMC in tumor cells with high or no COX-2 expression**. MIA-PaCa-2 (COX-2 negative) and Bx-PC-3 (high COX-2 expression) pancreatic carcinoma cells were treated with the indicated concentrations of CXB, UMC, or DMC for 18 hours and cell lysates were analyzed by Western blot with specific antibodies to CHOP (a pro-apoptotic ER stress indicator protein) and actin (to verify equal loading in each lane).

We next investigated the expression levels of the ER stress marker CHOP in response to drug treatment of MIA-PaCa-2 and Bx-PC-3 cells. Here as well, no major differences could be found between these COX-2-positive and COX-2-negative cells. As shown in Figure 6, 60 μM DMC and 75 μM CXB similarly increased CHOP protein levels, whereas 75 μM UMC had no major effect. This finding further indicated that induction of ER stress by celecoxib was independent of any involvement of COX-2.

**Figure 7:**
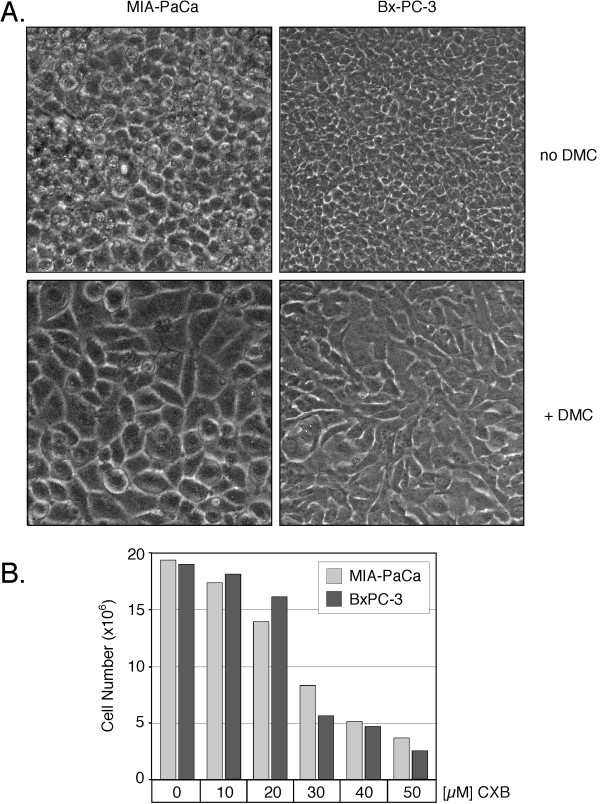
**Lower cell densities in the presence of CXB, DMC, and UMC**. MIA-PaCa-2 (COX-2 negative) and Bx-PC-3 (high COX-2 expression) pancreatic carcinoma cells were continuously exposed to various concentrations of CXB, DMC, or UMC for up to 2 months in the same cell culture dishes (i.e., without splitting the cell monolayers). In A., cells were treated with or without 20 μM DMC and photomicrographs (400× magnification) were taken after 6 weeks. Note that in the presence of drug (lower panels) the individual cells are noticeably larger, and the overall monolayer consists of fewer cells per surface area. In B., cells were treated with increasing concentrations of CXB and the total number of cells per well (6-well plate) was determined after 4 weeks (shown is the average of two counts). In all instances, the phenotypic changes were similar in the case of CXB, UMC, or DMC (not shown for all treatment conditions), except that DMC was the most potent, and UMC the least potent, compound. These experiments were repeated with very similar outcomes.

We also intended to perform focus formation assays with the MIA-PaCa-2 and Bx-PC-3 pair of cell lines. However, it turned out that these cells were unable to form the type of foci typical of many transformed cells. Instead, after forming a densely packed monolayer, these cells kept proliferating and shed the newly generated cells floating into the medium. However, in the continuous presence of CXB, DMC, or UMC, two phenotypic changes became apparent in both cell lines. First, after the completion of the monolayer, cell proliferation was greatly reduced and substantially fewer cells were shed into the medium, i.e., contact inhibition appeared to have been restored by drug treatment. Second, the confluent monolayer was constituted of larger cells, i.e., there were noticeably fewer cells per surface area and the cells appeared much less crowded in the presence of drugs (Figure 7). These phenotypic features were quite stable: even after extended periods of drug exposure (up to 2 months), drug-treated cell cultures did not display the compactness and density of untreated control cells. However, substantial differences in drug potency were noted. As before, DMC was the most potent compound, CXB was 20–30% less effective, and UMC clearly was the least effective of these drugs. Taken together, these results demonstrate that the presence or absence of COX-2 had no bearing on the phenotypic responses of these cells to drug treatment, indicating that CXB achieved these outcomes independently of any COX-2 involvement.

All of the above-described phenotypic changes were stable as long as drug treatment was continued, but were fully reversible after the removal of drugs (not shown). For instance, when drug treatment of MIA-PaCa-2 and Bx-PC-3 cells was discontinued after 2 months, the cell cultures became more crowded, i.e., the cell number per square inch increased and the monolayer resumed shedding of new cells into the culture medium; similarly, when drug-treated U251 monolayers, which had remained focus-free during 3 months of drug treatment, were trypsinized and freshly seeded into new culture dishes, they promptly developed foci in the absence of drug treatment (not shown). Thus, suppression of the transformed phenotype in these tumor cells was dependent on the continuous presence of sub-toxic concentrations of CXB, UMC, or DMC.

## Discussion

The question as to whether the antitumor effects of CXB are based on its famed ability to inhibit COX-2 has incited substantial controversy. This issue is particularly relevant in view of the life-threatening complications that have emerged during the long-term treatment with elevated dosages of this drug for chemopreventive or cancer therapeutic applications. These unwanted side effects, such as cardiovascular events, are thought to be due to the long-term inhibition of COX-2 and have also been observed with other coxibs, such as rofecoxib (Vioxx) [7]. In this regard, one might be tempted to speculate that analogs of CXB that are devoid of COX-2-inhibitory ability, yet maintain antitumor potency, might potentially be superior for certain cancer therapeutic purposes [27].

In order to further investigate the relevance of COX-2 for CXB's antitumor efficacy, we applied two close structural analogs of this drug that differentially display COX-2-inhibitory activity. UMC has maintained COX-2-inhibitory function and is even 20% more potent than its parental compound; in contrast, DMC is devoid of any substantial COX-2-inhibitory function. These analogs originally were generated by Penning et al. [23] as members of a large group of molecules that were screened for selective COX-2-inhibitory activity, an effort that ultimately led to the discovery of CXB. Several years later, this number of compounds was investigated by Chen's group at Ohio State University for their ability to induce apoptosis in cultured prostate cancer cells. In these studies [28,29], it was found that some of these compounds were able to induce tumor cell death in vitro, but that this pharmacologic activity did not correlate with the ability of the individual analogs to inhibit COX-2. Thus, it was concluded that apoptosis-inducing potency and COX-2-inhibitory ability resided in different parts of the CXB molecule and could be separated.

While much subsequent effort has been spent on fine-tuning and improving the COX-2-independent acute cytotoxicity of CXB analogs [27,30-33], essentially nothing is known about the long-term effects of sub-toxic concentrations of these compounds and whether or not the inhibition of COX-2 might emerge as a critical factor during extended drug exposure. For this reason, we applied UMC and DMC, which separately represent the two major characterized functions of CXB, namely inhibition of COX-2 (preserved at increased potency in UMC) and induction of apoptosis (preserved at increased potency in DMC).

Our results show that the IC50 for inhibition of COX-2 by CXB or UMC in cell culture is below 1 μM, and 10 μM of these drugs suffice to completely block COX-2 activity. These results are in agreement with earlier studies reporting potent inhibition of PGE_2 _synthesis in cell culture by submicromolar concentrations of CXB [34,35]. Intriguingly, at these low concentrations, neither CXB nor UMC generated any detectable consequences for the cellular phenotype. In particular, during short-term exposure, there was no inhibition of cell proliferation, no increased cytotoxicity, and no indication of ER stress (Figures 2, 3, 5 and 6); furthermore, during long-term exposure, these concentrations were unable to prevent focus formation (Figure 4) and did not reduce the compaction of cellular monolayers (Figure 7). We therefore conclude that mere inhibition of COX-2 does not impinge on tumor cell growth and survival, and does not impede focus formation or high cell densities, even during very long incubation times.

In contrast to low concentrations (≤10 μM) of CXB and UMC, further elevated concentrations of these compounds began to generate observable changes in tumor phenotype. However, it is noteworthy that UMC was substantially less effective than CXB in all these assays. Because UMC is no less potent than CXB when it comes to the inhibition of COX-2 and the reduction of cellular PGE_2 _levels, it would be expected that any cellular phenotype that is triggered by or dependent on the inhibition of COX-2 should become apparent at similar concentrations of these two drugs. As this clearly is not the case, these findings further support the conclusion that COX-2 has no role in the antitumor effects we have measured in our study.

DMC, which lacks substantial COX-2-inhibitory function, displayed the most potent antitumor effects in our study. The acute anti-proliferative and cytotoxic effects of this compound have been investigated before and have helped establish ER stress as potentially the most important mechanism by which this drug – and CXB as well – exert their cytotoxic effects [12,31,36,37]. Importantly, in vivo studies verified that ER stress-mediated cytotoxicity could also be achieved in experimental tumors of mice that were fed CXB or DMC in their chow [12]. These latter results helped refute earlier criticisms [38,39] that COX-2-independent effects of CXB might be artifacts of the in vitro culture system, a contention that was based on the observation that the drug concentrations required to affect most non-COX-2 targets in vitro were not achievable in patients or animals. Although true that the induction of ER stress in cell culture generally requires CXB or DMC concentrations of ≥40 or ≥30 μM, respectively [12,17-19], both drugs are able to trigger ER stress also in animal tumors in vivo [12,19], where drug concentrations measured in the serum are below 10 μM [12,37]. Although a solution to the conundrum of these differential drug concentration requirements has yet to be provided, these types of experiments nonetheless strongly caution against the tendency to minimize results that were obtained with the use of moderately high micromolar concentrations of celecoxib and DMC in vitro.

The vast majority of previous studies that investigated antitumor mechanisms of CXB in vitro applied drug concentrations that proved cytotoxic, i.e., CXB concentrations were increased to a level that effected proliferation-inhibitory and apoptosis-inducing outcomes within 24 to 96 hours. We reasoned that these acutely toxic effects of CXB, which were mostly interpreted as being COX-2 independent, perhaps might obscure other, potentially COX-2 dependent, effects that otherwise might emerge only when cells do not undergo immediate cell death. For this reason, we applied CXB, UMC, and DMC at sub-toxic concentrations that did not trigger ER stress and subsequent apoptosis. Under these much longer-term conditions, a new feature of these drugs emerged, namely the ability to restore contact inhibition and block focus formation of tumor cells (Figures 4 and 7).

The predisposition to form foci is a characteristic of many cancer cells and indicates their acquired ability to disregard the growth-inhibitory signals emanating from the close contact with neighboring cells. This in vitro feature is thought to reflect the in vivo ability of tumor cells to escape social constraints within their tissue of origin and might represent an important prerequisite for the initiation of tumor growth [40]. In this context, it is tempting to speculate that the ability of CXB to suppress focus formation perhaps might represent a component of this drug's recognized chemopreventive potential. However, our results were obtained with the use of fully transformed, malignant tumor cells in vitro, which represents a poor model to study chemoprevention; therefore, better models will be needed to further investigate our conjecture. Similarly, with regards to therapeutic applications for advanced cancers, CXB's ability to restore contact inhibition conceivably could contribute to the reduced growth of established tumors that has been reported in many animal tumor models treated with this drug (selected examples: [2,37,39,41]).

## Conclusion

Our study shows that COX-2 inhibition by CXB has no detectable consequences for various molecular and cellular markers of tumor growth in several glioblastoma and pancreatic carcinoma cell lines with variable levels of COX-2 expression. Inhibition of COX-2 per se is neither required nor sufficient to implement the acutely growth-inhibitory and cytotoxic effects of celecoxib, nor does it impinge on the focus-forming ability of tumor cells during chronic exposure at sub-toxic concentrations. Consistent with several earlier reports [11,12,17-19], the observed short-term antitumor effect of CXB closely correlates with this drug's ability to trigger ER stress, and this mechanism provides a reasonable explanation for the drug's acutely growth-inhibitory effects. However, CXB's potency to restore contact inhibition of tumor cells takes place at drug concentrations that neither trigger substantial ER stress nor cause cytotoxicity, and also cannot be explained by the inhibition of COX-2. It therefore appears that additional, as yet uncharacterized, molecular mechanisms of this drug are at work during long-term, chronic treatment of tumor cells. Considering that the envisioned application of CXB for chemopreventive or therapeutic anticancer purposes will require long-term drug exposure of patients, it will be important to determine the molecular mechanisms that underlie this newly recognized ability of CXB to suppress focus formation during the chronic treatment of tumor cells.

## Materials and methods

### Materials

CXB was obtained as Celebrex^® ^capsules from the pharmacy, or was synthesized in our laboratory according to previously published procedures [23]. DMC was synthesized in our laboratory according to previously published procedures [31]. UMC as well was synthesized in our laboratory according to previously published procedures [23]. All compounds were dissolved in DMSO at 100 mM (stock solution) and added to the cell culture medium in a manner that kept the final concentration of solvent below 0.1%.

### Cell lines and culture conditions

The following human tumor cell lines were used: U251, LN229, and T98G glioblastoma (kindly provided by Dr. Frank B. Furnari, Ludwig Institute of Cancer Research, La Jolla, CA) and MIA PaCa-2 and BxPC-3 pancreatic carcinoma (kindly provided by Dr. Guido Eibl, UCLA, Los Angeles, CA). All cells were propagated in DMEM (GIBCO BRL, Grand Island, NY) supplemented with 10% fetal bovine serum, 100 U/ml penicillin, and 0.1 mg/ml streptomycin in a humidified incubator at 37°C and a 5% CO_2 _atmosphere.

### MTT assays

MTT assays were performed in 96-well plates as described in detail elsewhere [36]. All assays were repeated several times at variable cell densities. The number of cells per well ranged from 3.0 × 10^3 ^to 8.0 × 10^3^.

### Immunoblots

Total cell lysates were prepared and analyzed by Western blot analysis with two different procedures. One was based on conventional enzyme-linked chemoluminescence with HRP-conjugated secondary antibodies as described earlier [31], the other used the Odyssey infrared imaging system (LI-COR Biosciences, Lincoln, NE) and fluorescence-conjugated secondary antibodies, according to protocols supplied by the manufacturer. In both cases, the primary antibodies were from Cell Signaling Technologies (Beverly, MA) or from Santa Cruz Biotechnology, Inc. (Santa Cruz, CA) and were used according to the manufacturers' recommendations. All immunoblots were repeated at least once with new lysates from a duplicate experiment to confirm the results.

### Focus formation assays

Cells were seeded into 6-well plates at low density (approximately 20% confluence). One day later, increasing concentrations of celecoxib, DMC, or UMC were added. After 4–5 days, untreated control cells had covered the entire surface area of the well and began to form a monolayer that became more compact over time. From this time on, the growth medium and drugs were replaced every two days. After approximately 10 days, focus formation became noticeable and was quantitated by counting the number of foci per microscopic field every ten days. In addition, digital images were captured with a SPOT camera model 1.4.0 operated with Spot Advanced Software version 4.0.8 (Diagnostic Instruments, Sterling Heights, MI) and saved in 12-bit grayscale format with 1600 x 1200 active pixels.

## Authors' contributions

H-CC and AK performed experiments. KJG and NAP were responsible for synthesizing the various drugs. AHS conceived of the study and directed it. All authors read and approved of the final manuscript.

## References

[B1] Steinbach G, Lynch PM, Phillips RK, Wallace MH, Hawk E, Gordon GB, Wakabayashi N, Saunders B, Shen Y, Fujimura T (2000). The effect of celecoxib, a cyclooxygenase-2 inhibitor, in familial adenomatous polyposis. N Engl J Med.

[B2] Masferrer JL, Leahy KM, Koki AT, Zweifel BS, Settle SL, Woerner BM, Edwards DA, Flickinger AG, Moore RJ, Seibert K (2000). Antiangiogenic and antitumor activities of cyclooxygenase-2 inhibitors. Cancer Res.

[B3] Dannenberg AJ, Subbaramaiah K (2003). Targeting cyclooxygenase-2 in human neoplasia: rationale and promise. Cancer Cell.

[B4] Koehne CH, Dubois RN (2004). COX-2 inhibition and colorectal cancer. Semin Oncol.

[B5] Arber N, Eagle CJ, Spicak J, Racz I, Dite P, Hajer J, Zavoral M, Lechuga MJ, Gerletti P, Tang J (2006). Celecoxib for the prevention of colorectal adenomatous polyps. N Engl J Med.

[B6] Bertagnolli MM, Eagle CJ, Zauber AG, Redston M, Solomon SD, Kim K, Tang J, Rosenstein RB, Wittes J, Corle D (2006). Celecoxib for the prevention of sporadic colorectal adenomas. N Engl J Med.

[B7] Grosser T, Fries S, FitzGerald GA (2006). Biological basis for the cardiovascular consequences of COX-2 inhibition: therapeutic challenges and opportunities. J Clin Invest.

[B8] Grösch S, Maier TJ, Schiffmann S, Geisslinger G (2006). Cyclooxygenase-2 (COX-2)-independent anticarcinogenic effects of selective COX-2 inhibitors. J Natl Cancer Inst.

[B9] Kashfi K, Rigas B (2005). Non-COX-2 targets and cancer: Expanding the molecular target repertoire of chemoprevention. Biochem Pharmacol.

[B10] Schönthal AH (2007). Direct non-cyclooxygenase-2 targets of celecoxib and their potential relevance for cancer therapy. Br J Cancer.

[B11] Johnson AJ, Hsu AL, Lin HP, Song X, Chen CS (2002). The cyclo-oxygenase-2 inhibitor celecoxib perturbs intracellular calcium by inhibiting endoplasmic reticulum Ca2+-ATPases: a plausible link with its anti-tumour effect and cardiovascular risks. Biochem J.

[B12] Pyrko P, Kardosh A, Liu YT, Soriano N, Xiong W, Chow RH, Uddin J, Petasis NA, Mircheff AK, Farley RA (2007). Calcium-activated ER stress as a major component of tumor cell death induced by 2,5-dimethyl-celecoxib (DMC), a non-coxib analog of celecoxib. Mol Cancer Ther.

[B13] Tanaka K, Tomisato W, Hoshino T, Ishihara T, Namba T, Aburaya M, Katsu T, Suzuki K, Tsutsumi S, Mizushima T (2005). Involvement of intracellular Ca2+ levels in nonsteroidal anti-inflammatory drug-induced apoptosis. J Biol Chem.

[B14] Alloza I, Baxter A, Chen Q, Matthiesen R, Vandenbroeck K (2006). Celecoxib inhibits interleukin-12 alphabeta and beta2 folding and secretion by a novel COX2-independent mechanism involving chaperones of the endoplasmic reticulum. Mol Pharmacol.

[B15] Wang JL, Lin KL, Chen JS, Lu YC, Jiann BP, Chang HT, Hsu SS, Chen WC, Huang JK, Ho CM, Jan CR (2004). Effect of celecoxib on Ca2+ movement and cell proliferation in human osteoblasts. Biochem Pharmacol.

[B16] Kim SH, Hwang CI, Park WY, Lee JH, Song YS (2007). GADD153 mediates celecoxib-induced apoptosis in cervical cancer cells. Carcinogenesis.

[B17] Pyrko P, Kardosh A, Schönthal AH (2008). Celecoxib transiently inhibits protein synthesis. Biochem Pharmacol.

[B18] Tsutsumi S, Gotoh T, Tomisato W, Mima S, Hoshino T, Hwang HJ, Takenaka H, Tsuchiya T, Mori M, Mizushima T (2004). Endoplasmic reticulum stress response is involved in nonsteroidal anti-inflammatory drug-induced apoptosis. Cell Death Differ.

[B19] Tsutsumi S, Namba T, Tanaka KI, Arai Y, Ishihara T, Aburaya M, Mima S, Hoshino T, Mizushima T (2006). Celecoxib upregulates endoplasmic reticulum chaperones that inhibit celecoxib-induced apoptosis in human gastric cells. Oncogene.

[B20] Bundscherer A, Hafner C, Maisch T, Becker B, Landthaler M, Vogt T (2008). Antiproliferative and proapoptotic effects of rapamycin and celecoxib in malignant melanoma cell lines. Oncol Rep.

[B21] Ko SH, Choi GJ, Lee JH, Han YA, Lim SJ, Kim SH (2008). Differential effects of selective cyclooxygenase-2 inhibitors in inhibiting proliferation and induction of apoptosis in oral squamous cell carcinoma. Oncol Rep.

[B22] Kashfi K, Rigas B (2005). Is COX-2 a 'collateral' target in cancer prevention?. Biochem Soc Trans.

[B23] Penning TD, Talley JJ, Bertenshaw SR, Carter JS, Collins PW, Docter S, Graneto MJ, Lee LF, Malecha JW, Miyashiro JM (8635). Synthesis and biological evaluation of the 1,5-diarylpyrazole class of cyclooxygenase-2 inhibitors: identification of 4-[5-(4-methylphenyl)-3-(trifluoromethyl)-1H-pyrazol-1-yl]benze nesulfonamide (SC-5 celecoxib). J Med Chem.

[B24] Kardosh A, Blumenthal M, Wang WJ, Chen TC, Schönthal AH (2004). Differential effects of selective COX-2 inhibitors on cell cycle regulation and proliferation of glioblastoma cell lines. Cancer Biol Ther.

[B25] Eibl G, Bruemmer D, Okada Y, Duffy JP, Law RE, Reber HA, Hines OJ (2003). PGE(2) is generated by specific COX-2 activity and increases VEGF production in COX-2-expressing human pancreatic cancer cells. Biochem Biophys Res Commun.

[B26] Liu Y-T, Kardosh A, Cooc J, Schönthal AH (2006). Potential misidentification of cyclooxygenase-2 by Western blot analysis and prevention through the inclusion of appropriate controls. Molecular Biotechnology.

[B27] Schönthal AH, Chen TC, Hofman FM, Louie SG, Petasis NA (2008). Celecoxib analogs that lack COX-2 inhibitory function: preclinical development of novel anticancer drugs. Expert Opin Investig Drugs.

[B28] Song X, Lin HP, Johnson AJ, Tseng PH, Yang YT, Kulp SK, Chen CS (2002). Cyclooxygenase-2, player or spectator in cyclooxygenase-2 inhibitor-induced apoptosis in prostate cancer cells. J Natl Cancer Inst.

[B29] Zhu J, Song X, Lin HP, Young DC, Yan S, Marquez VE, Chen CS (2002). Using cyclooxygenase-2 inhibitors as molecular platforms to develop a new class of apoptosis-inducing agents. J Natl Cancer Inst.

[B30] Ding H, Han C, Zhu J, Chen CS, D'Ambrosio SM (2005). Celecoxib derivatives induce apoptosis via the disruption of mitochondrial membrane potential and activation of caspase 9. Int J Cancer.

[B31] Kardosh A, Wang W, Uddin J, Petasis NA, Hofman F, Chen CC, Schönthal AH (2005). Dimethyl-celecoxib (DMC), a derivative of celecoxib that lacks cyclooxygenase-2-inhibitory function, potently mimics the anti-tumor effects of celecoxib on Burkitt's lymphoma in vitro and in vivo. Cancer Biol Ther.

[B32] Kusunoki N, Ito T, Sakurai N, Handa H, Kawai S (2006). A celecoxib derivative potently inhibits proliferation of colon adenocarcinoma cells by induction of apoptosis. Anticancer Res.

[B33] Zhu J, Huang JW, Tseng PH, Yang YT, Fowble J, Shiau CW, Shaw YJ, Kulp SK, Chen CS (2004). From the cyclooxygenase-2 inhibitor celecoxib to a novel class of 3-phosphoinositide-dependent protein kinase-1 inhibitors. Cancer Res.

[B34] Kim SH, Song SH, Kim SG, Chun KS, Lim SY, Na HK, Kim JW, Surh YJ, Bang YJ, Song YS (2004). Celecoxib induces apoptosis in cervical cancer cells independent of cyclooxygenase using NF-kappaB as a possible target. J Cancer Res Clin Oncol.

[B35] Schroeder CP, Yang P, Newman RA, Lotan R (2004). Eicosanoid metabolism in squamous cell carcinoma cell lines derived from primary and metastatic head and neck cancer and its modulation by celecoxib. Cancer Biol Ther.

[B36] Kardosh A, Soriano N, Liu Y-T, Uddin J, Petasis NA, Hofman F, Chen CC, Schönthal AH (2005). Multi-target inhibition of drug-resistant multiple myeloma cell lines by dimethyl-celecoxib (DMC), a non-COX-2-inhibitory analog of celecoxib. Blood.

[B37] Kulp SK, Yang YT, Hung CC, Chen KF, Lai JP, Tseng PH, Fowble JW, Ward PJ, Chen CS (2004). 3-phosphoinositide-dependent protein kinase-1/Akt signaling represents a major cyclooxygenase-2-independent target for celecoxib in prostate cancer cells. Cancer Res.

[B38] Raz A (2002). Is inhibition of cyclooxygenase required for the anti-tumorigenic effects of nonsteroidal, anti-inflammatory drugs (NSAIDs)? In vitro versus in vivo results and the relevance for the prevention and treatment of cancer. Biochem Pharmacol.

[B39] Williams CS, Watson AJ, Sheng H, Helou R, Shao J, DuBois RN (2000). Celecoxib prevents tumor growth in vivo without toxicity to normal gut: lack of correlation between in vitro and in vivo models. Cancer Res.

[B40] Alberts B, Johnson A, Lewis J, Raff M, Rogerts K, Walter P (2002). Molecular Biology of the Cell.

[B41] Roh JL, Sung MW, Park SW, Heo DS, Lee DW, Kim KH (2004). Celecoxib can prevent tumor growth and distant metastasis in postoperative setting. Cancer Res.

[B42] Schönthal AH (2006). Antitumor properties of dimethyl-celecoxib, a derivative of celecoxib that does not inhibit cyclooxygenase-2: implications for glioblastoma therapy. Neurosurgical Focus.

